# Supplementary material for: White matter maturation is associated with the emergence of Theory of Mind in early childhood
Source: Nat Commun. 2017 Mar 21;8:14692. doi: 10.1038/ncomms14692 (PMC5364393; doi:10.1038/ncomms14692)
Supplement: Supplementary Information — Supplementary Figure, Supplementary Tables, Supplementary Methods and Supplementary References [file ncomms14692-s1.pdf]

Supplementary Table 1: Variance in the false belief score explained by age-related changes in FA according to commonality analysis

**Region: WM in / near**	**Explained variance**	**(% Overlap)**	**p-value**
rTPJ	7.4 %	(95)	10^{-12}
rpMTG/TPJ	6.8 %	(90)	.005
lMTG	7.5 %	(98)	.0007
lITG	6.2 %	(92)	.0004
lSTG	6.4 %	(94)	.01
rvMPFC	4.3 %	(71)	.03
rSPL/PC	6.5 %	(97)	.01
lThalamus	9.8 %	(100)	.0004

WM – white matter, r – right, l – left, p – posterior, TPJ – temporoparietal junction, MTG – middle temporal gyrus, ITG – inferior temporal gyrus, STG – superior temporal gyrus, vMPFC – ventromedial prefrontal cortex, SPL – superior parietal lobule, PC – precuneus.

Mean explained variance in the false belief score by the common contributions of age and FA, while language, executive function, and implicit belief-related anticipation scores were controlled for. The mean is computed across significant voxels of the regions that correlated with the false belief scores in the TBSS analysis. The reported effects were significant at $p < .01$ tested against a random baseline with 5000 permutations at voxel-level and corrected for cluster size (p-values two-sided). The overlap, i.e., the percentage of voxels with significant contribution in the commonality analysis from all voxels in the TBSS region, is reported in brackets

Supplementary Table 2: TBSS results when scoring only the first question of each of the false belief tasks

WM in / near	MNI coordinates CoG			Size in voxels	Correlation coefficient r
rTPJ	32	-45	30	49	.44
rpMTG/TPJ	42	-48	16	39	.42
lMTG	-40	-20	-12	39	.46
lITG	-42	-34	-6	13	.50
lSTG	-32	-30	5	27	.41
rvMPFC	25	37	1	24	.42

WM – white matter, MNI – Montreal Neurological Institute, CoG – center of gravity, r – right, l – left, p – posterior, a – anterior, TPJ – temporoparietal junction, MTG – middle temporal gyrus, ITG – inferior temporal gyrus, STG – superior temporal gyrus, vMPFC – ventromedial prefrontal cortex, SPL – superior parietal lobule, PC – precuneus, AF – arcuate fascicle, ECFS – extreme capsule fiber system, ILF – inferior longitudinal fascicle, IFOF – inferior fronto-occipital fascicle, CC – corpus callosum, TR – thalamic radiation.

Supplementary Table 3: Correlation with streamline density when scoring only the first question of each of the false belief tasks.

WM tract	MNI coordinates CoG			Size in voxels	Correlation coefficient r
rAF	28	33	14	90	.39
rAF	28	25	24	151	.39
rAF	26	1	31	346	.38
lAF	-25	30	14	65	.40
rIFOF	33	-5	-14	155	.40

r – right, l – left, AF – arcuate fascicle, IFOF – inferior fronto-occipital fascicle, IFG – inferior frontal gyrus, MTG – middle temporal gyrus.

Supplementary Figure 1

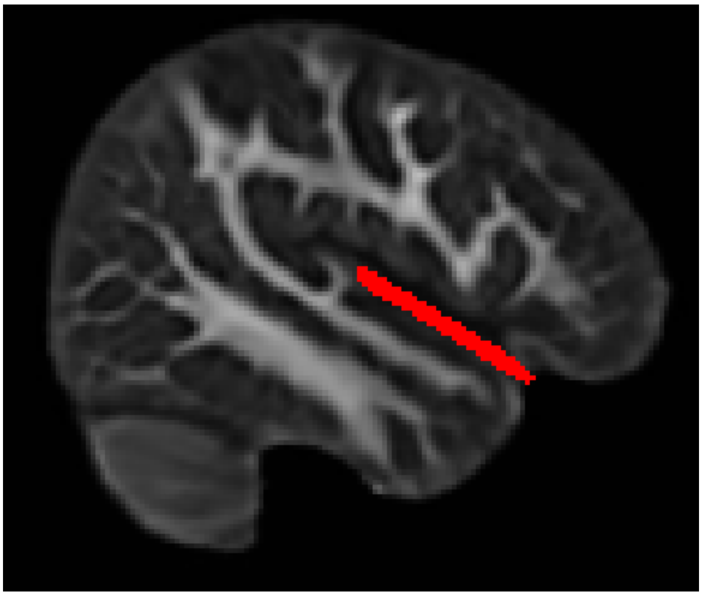

Termination mask for an additional tractography restricted to dorsal pathways. A plane parallel to the Sylvian fissure was defined as a termination mask for an additional probabilistic tractography from the seed regions in the left MTG and right TPJ. This confirmed that the observed significant effect in the IFG stemmed from dorsal and not ventral streamlines.

Supplementary Methods:

TBSS Analyses for Other Cognitive Domains

Similar to the TBSS analysis for the explicit false belief score, a TBSS analysis was performed to assess the correlation of FA with the executive function score, the standardized language test, and the implicit belief-related anticipatory looking task. Additionally, as for the false belief score, a voxelwise commonality analysis was performed on the skeleton including age and FA as predictors for the dependent variable of interest to test whether the effects were age-related.

Executive functions. A TBSS analysis revealed a significant correlation of the executive function score with FA in the left lingual gyrus (O5) (MNI coordinates center of gravity (CoG): x = -22, y = -65, z = 0), which has been shown to be involved in attentional modulation towards different visual features of objects[1] and in categorization[2]. A second effect was found with FA in the hand area of the right postcentral gyrus (S1) (MNI coordinates CoG: x = 37, y = -29, z = 51). The TBSS commonality analysis showed that the effects both stemmed from age-related changes in FA: The shared contribution of age and FA in O5 explained 8% and of age and FA in S1 explained 9.6% of variance in the false belief score.

Language. For language, a TBSS analysis yielded one effect in the white matter bordering the right IFG (MNI coordinates CoG: x = 34, y = 25, z = 31) which proved to be age-independent in the commonality analysis (unique contribution of FA in right IFG: 21.4%; contributions including age: not significant). Two further clusters made no significant contribution in the commonality analysis. Broca's area in the IFG is known to support language processing bilaterally in early childhood with increasing left lateralization in the course of development[3].

Belief-related anticipation. A TBSS analysis of the correlation of FA with the percent correct anticipatory looking in the FB trials of the implicit belief-related anticipatory looking task revealed no significant correlation of FA and implicit belief-related anticipation. Future research should follow up on the brain regions and connections relevant for mastering implicit false belief tasks, possibly using a battery of different tasks including anticipatory looking as well as violation

of expectation paradigms. Such an approach should also study younger infants at an age when the ability emerges and an age-difference in performance can be observed.

Supplementary References

1. Corbetta M., Miezin F.M., Dobmeyer S., Shulman G.L., & Petersen S.E. Attentional modulation of neural processing of shape, color, and velocity in humans. *Science* **22**, 1556–1559 (1990).
2. Devlin J.T., et al. Is there an anatomical basis for category-specificity? Semantic memory studies in PET and fMRI. *Neuropsychologia* **40**, 54–75 (2002).
3. Friederici A.D., Brauer J., Lohmann G. Maturation of the Language Network: From Inter- to Intrahemispheric Connectivities. *PLoS ONE* **6(6)**, doi:10.1371/journal.pone.0020726 (2011).
